# Treatment of pro-B acute lymphoblastic leukemia and severe plaque psoriasis with anti-CD19 CAR T cells: a case report

**DOI:** 10.3389/fimmu.2025.1529745

**Published:** 2025-03-03

**Authors:** Ao Zhang, Guangji Zhang, Huihui Yang, Bengfa Gong, Shouyun Li, Na Wei, Hui Xue, Hui Wei, Jianxiang Wang, Shaowei Qiu

**Affiliations:** ^1^ State Key Laboratory of Experimental Hematology, National Clinical Research Center for Blood Diseases, Haihe Laboratory of Cell Ecosystem, Institute of Hematology and Blood Diseases Hospital, Chinese Academy of Medical Sciences and Peking Union Medical College, Tianjin, China; ^2^ Tianjin Institutes of Health Science, Tianjin, China

**Keywords:** pro-B ALL, psoriasis, CD19 CAR T cells, B cell, therapeutic targets

## Abstract

We report on a rare case of adult pro-B acute lymphoblastic leukemia (pro-B ALL) accompanied with severe refractory plaque psoriasis treated using autologous anti-CD19 chimeric antigen receptor (CAR) T-cell therapy. An 18-year-old man with a known history of mild plaque psoriasis for 1 year was diagnosed with pro-B ALL. After induction chemotherapy, his psoriasis began to worsen. Extensive erythema and desquamation developed on the whole body, with severe plaque psoriasis on the knees and elbows, which did not respond to topical therapy. The application of CAR T-cell therapy not only enabled the patient to achieve deep complete remission (CR) but also allowed his skin lesions to completely subside. This successful treatment supports a potential pathogenic link between B cells and psoriasis, which could provide a new option for overcoming refractory psoriasis.

## Introduction

Psoriasis is a common chronic papulosquamous skin disease classified as an inflammatory disease. Its most common form, chronic plaque psoriasis or psoriasis vulgaris, presents with demarcated plaques covered in silvery scales. The variable size and thickness of the plaques reflect disease activity and treatment response. Mild plaque psoriasis could benefit from topical therapy; for moderate-to-severe psoriasis, biologics, oral agents, or phototherapies should be considered (1). The core pathogenesis of psoriasis involves T-cell-dependent immune responses. Therefore, a few research studies have focused on whether B-cell-targeting therapy could be used. In this paper, we reported on a rare case of adult pro-B acute lymphoblastic leukemia (pro-B ALL) accompanied with severe refractory plaque psoriasis. The application of autologous anti-CD19 chimeric antigen receptor (CAR) T-cell therapy not only enabled the patient to achieve deep complete remission (CR) but also allowed his skin lesions to completely subside.

## Case presentation

**Figure 1:**
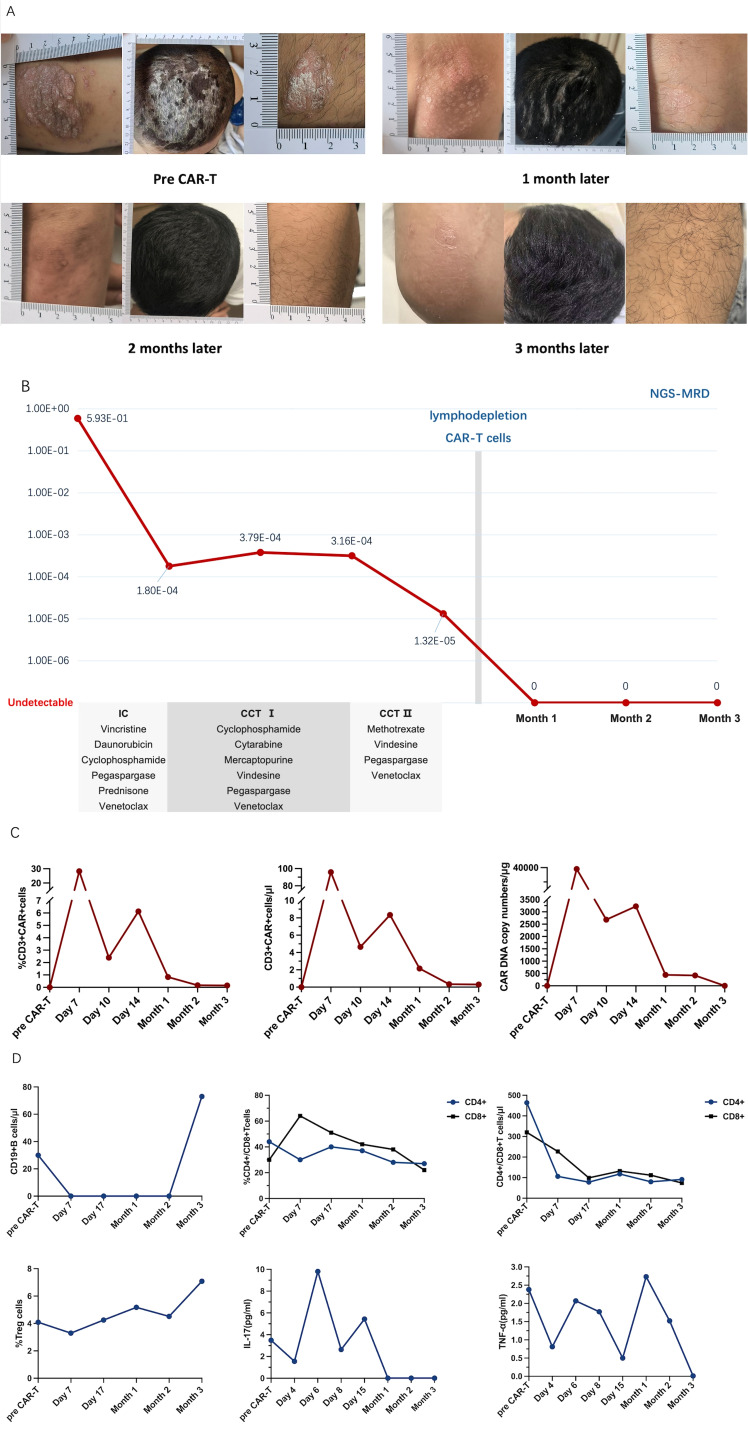
**(A)** Extensive erythema and desquamation on the whole body, especially on the scalp, with severe plaque psoriasis on the left elbow and right knee at baseline and after chimeric antigen receptor (CAR) T-cell infusion. **(B)** Timeline of the treatment including one course of induction chemotherapy (IC) and two courses of consolidation chemotherapy (CCT) before CAR T-cell therapy. The effect was reflected using a more sensitive immunoglobulin heavy chain next-generation sequencing for minimal residual disease (IgH NGS-MRD). **(C)** Peripheral CD3^+^ CAR T-cell concentration (in percent and in cells per microliter) and CAR DNA copy number (in genome per microgram) after CAR T-cell infusion by flow cytometry. **(D)** B-cell counts, CD4^+^ and CD8^+^ T-cell concentrations (in percent and in cells per microliter), and circulating regulatory T-cell proportions along with interleukin 17 (IL-17) and anti-tumor necrosis factor alpha (TNF-α) levels at baseline and after CAR T-cell infusion.

An 18-year-old man, who was diagnosed with plaque psoriasis in 2022, presented with mild thickness and desquamation on the surface of the elbow. Topical therapy effectively improved his skin lesions. At the end of 2023, he was diagnosed with pro-B ALL. After induction chemotherapy, his psoriasis began to worsen. Extensive erythema and desquamation developed on the whole body, with severe plaque psoriasis on the knees and elbows, which did not respond to topical therapy (**Figure 1A**). Despite the worsening of his psoriasis symptoms, no psoriasis-related complications were observed. Unfortunately, the patient failed to achieve durable deep remission after several courses of chemotherapy (**Supplementary Table S1**). This implied the need for allograft transplant. However, the patient refused due to concerns of transplant complications. The patient consequently received anti-CD19 CAR T-cell therapy to eradicate residual blast cells. He underwent lymphodepleting chemotherapy with fludarabine (30 mg/m^2^ per day) and cyclophosphamide (0.3 g/m^2^ per day) for three consecutive days before the administration of 2 × 10^6^ cells/kg autologous CD19 CAR T cells. The patient developed a fever on day 4 after anti-CD19 CAR T-cell therapy, with a temperature reaching 39°C. This was considered grade 1 cytokine release syndrome (CRS). The condition of the patient improved after treatment with cephalosporin antibiotics and dexamethasone. No other adverse events were observed after this episode.

To our surprise, the size and the thickness of the plaques greatly decreased, and the erythema faded noticeably. The psoriasis plaques and scalp lesions completely subsided 2 months after CAR T-cell infusion. The erythema was replaced by mild pigmentation (**Figure 1A**). The refractory residual blast leukemia cells were also completely eliminated, as reflected in sensitive immunoglobulin heavy chain (IgH) next-generation sequencing (NGS) (**Figure 1B**). The CAR T cells proliferated well and were monitored continuously (**Figure 1C**). CD19^+^ B cells were depleted by day 7 and recovered on the third month. The proportions of CD4^+^ and CD8^+^ T cells were gradually reduced and that of regulatory T cells (Tregs) remained consistent, while the serum levels of interleukin 17 (IL-17) and tumor necrosis factor alpha (TNF-α) fluctuated (**Figure 1D**). Data on the levels of the complement components 3 and 4, interferon-α, interferon-γ, and immunoglobulins A and G before and after CAR T-cell infusion are presented in the **Supplementary Appendix**. To date, the patient has remained free from any recurrence of psoriasis, and all of his hematological parameters have remained within normal limits.

## Discussion

In recent years, CAR T-cell therapy has been the most rapidly developed and widely applied type of cellular immunotherapy due to its ability for deep B-cell depletion. T lymphocytes engineered to express CARs could recognize and respond to specific B cells independently of a major histocompatibility complex (MHC) engagement. The most popular target of CAR T cells is CD19, with other classic targets including CD20, CD22, and B-cell maturation antigen (BCMA) (2). CAR T-cell therapy drastically changed the landscape of antitumor treatment, especially against hematological malignancies. In the last 2 years, this technology has been introduced for the treatment of autoimmune diseases such as systemic lupus erythematosus and rheumatoid arthritis, among others. As autoreactive B-cell clones and autoantibodies are directed against a patient’s own antigens, they play a key role in the pathogenesis of autoimmune diseases. However, psoriasis has not been classified into these due to the conventional wisdom being that psoriasis has no B-cell-mediated pathophysiology (3).

Psoriasis is traditionally viewed as an inflammatory cutaneous disease mediated by T-cell-dependent immune responses, with IL-17 and IL-23 as key drivers. TNF-α and interferons also participate in the cross talk between the innate and adaptive immune systems. Moreover, the so-called feed-forward amplification of psoriasis inflammation is one of the central mechanisms (1).

In this case, the patient had a 2-year history of plaque psoriasis prior to the diagnosis of B-ALL. After induction chemotherapy, his psoriasis began to worsen, whereas subsequent CD19 CAR T-cell therapy led to its complete resolution. Chemotherapy-induced toxicity or leukemia-associated stress could disrupt the immune homeostasis and contribute to psoriasis aggravation. Following the CAR T-cell therapy, the CD4^+^ and CD8^+^ T-cell counts in the patient’s blood gradually decreased, while the Treg frequency increased over time, potentially contributing to psoriasis remission by suppressing the inflammatory T-cell activity. The levels of IL-17 and TNF-α fluctuated, but showed an overall decline, correlating with the resolution of the lesion. These findings suggest that CAR T-cell therapy might indirectly modulate the pathogenic T-cell responses by eliminating the B-cell-mediated immune activation. However, as CAR T-cell therapy primarily targets B cells, further studies are required to determine whether the therapeutic efficacy is a result of B-cell depletion specifically or a broader immune reconstitution.

Currently, the biologics for psoriasis mainly target specific inflammatory mediators, particularly the dominant IL-23/Th17 axis. Although biologic therapies are considered the most effective treatment for psoriasis, their associated side effects could pose substantial challenges, frequently precipitating refractory episodes of the disease. In recent years, the involvement of B cells in the pathogenesis of psoriasis has started to gain increasing attention. IL-10, a key anti-inflammatory cytokine in psoriasis, regulates T-cell-mediated inflammation. The transcription factor NFATc1 in B cells suppresses IL-10 secretion, highlighting B cells as a potential therapeutic target for psoriasis (4). Alterations in the various B-cell subsets in psoriasis underscore the pivotal role of B cells in the pathogenesis of the disease (5). Elevated levels of CD19^+^ activated B cells have been linked to the severity of psoriasis (6). An increase in regulatory B cells suppressed the IL-23-mediated psoriasis-like inflammation through the expansion of Tregs and the inhibition of Th17 differentiation (7). Longitudinal tracking of B cells showed that isotype switching from IgG-expressing B cells might be the major source of IgE in psoriasis, which is closely associated with skin inflammation (8). Furthermore, recent studies have confirmed the essential role of autoantigens in psoriasis. Hence, B cells engaged in autoantigen-mediated immune responses drive inflammation, cellular damage, and tissue destruction (4). B cells also act as antigen-presenting cells (APCs) to enhance CD4^+^ T-cell responses.

It is worth mentioning that the first case of successful CAR T-cell therapy in a patient with refractory/relapsed diffuse large B-cell lymphoma and chronic generalized plaque psoriasis has been reported recently. This patient had been suffering from generalized plaque psoriasis for 45 years, and rituximab had no effect on his lesions, which may be explained by the persistence of autoreactive B cells in lymphatic organs and inflamed tissues or that CD20 might not be a good target (compared with CD19 therapy, CD20 therapy spares early B cells, minimizing its impact on B-cell reconstitution) (9). Furthermore, due to the unclear mechanisms of CAR T-cell therapy in psoriasis, its substantial adverse effects, and the high cost, as well as the overall efficacy of the existing biologics in most psoriasis cases, no additional cases of CAR T-cell treatment for psoriasis have been reported. Consequently, our understanding of the feasibility and the risks associated with CAR T-cell therapy for psoriasis remains limited. Nevertheless, this case provides a novel perspective on the potential therapeutic application of B-cell depletion in severe refractory psoriasis and underscores the need for further research into the role of B cells in the pathogenesis of psoriasis.

In this paper, we reported on a rare case of severe plaque psoriasis and pro-B ALL treated with upfront CD19 CAR T-cell therapy. The refractory residual blast leukemia cells were completely eliminated and the severe plaque psoriasis thoroughly faded, with the efficacy still evident up to the present. This successful treatment supports a potential pathogenic link between B cells and psoriasis, which could provide a new option for overcoming refractory psoriasis.

## Data Availability

The original contributions presented in the study are included in the article/**Supplementary Material**. Further inquiries can be directed to the corresponding author.
