# Supplementary material for: Treatment of pro-B acute lymphoblastic leukemia and severe plaque psoriasis with anti-CD19 CAR T cells: a case report
Source: Front Immunol. 2025 Mar 3;16:1529745. doi: 10.3389/fimmu.2025.1529745 (PMC11911368; doi:10.3389/fimmu.2025.1529745)
Supplement: Supplementary file 1 [file DataSheet1.docx]

**Supplementary appendix**

1. **diagnosis of pro B-ALL**

He was admitted with an outside diagnosis of acute leukemia (12% circulating blasts). Bone marrow aspiration revealed 96% blasts, positive for CD19, cCD79a, CD34, CD38, CD123, HLA-DR, CD22 and TdT. Cytochemical staining showed positive periodic acid-Schiff (100%). Cytogenetic analysis revealed an abnormal karyotype: 45, XY, -7[2]/46, XY [28]. Targeted sequencing showed the presence of IKZF1 and IDH1 mutations (p.R132C, VAF: 3.60%). Diagnosis of proB acute lymphoblastic leukemia with IKZF1, IDH2 mutation and chromosome 7 deletion was rendered.

1. **Past Medical History, Personal History, Family History, and Physical Examination Findings**

The patient is generally in good health, with no history of infectious diseases, hypertension, diabetes mellitus, hyperlipidemia, or cardiovascular and cerebrovascular diseases. The patient denies any psychiatric disorders, history of endemic diseases, occupational illnesses, trauma, poisoning, or surgical interventions, as well as any known drug or food allergies. The vaccination history is unclear, and there is no history of blood transfusions. The patient's lifestyle is relatively regular, with no reported exposure to chemicals, radiation, or toxic substances. There is no history of recreational drug use, though the patient has a smoking history of approximately 10 cigarettes per day and occasional alcohol consumption. There is no family history of hereditary diseases or similar conditions. On physical examination, scattered psoriatic plaques were noted on the skin, with no other remarkable findings.

1. **Supplementary figures**

**
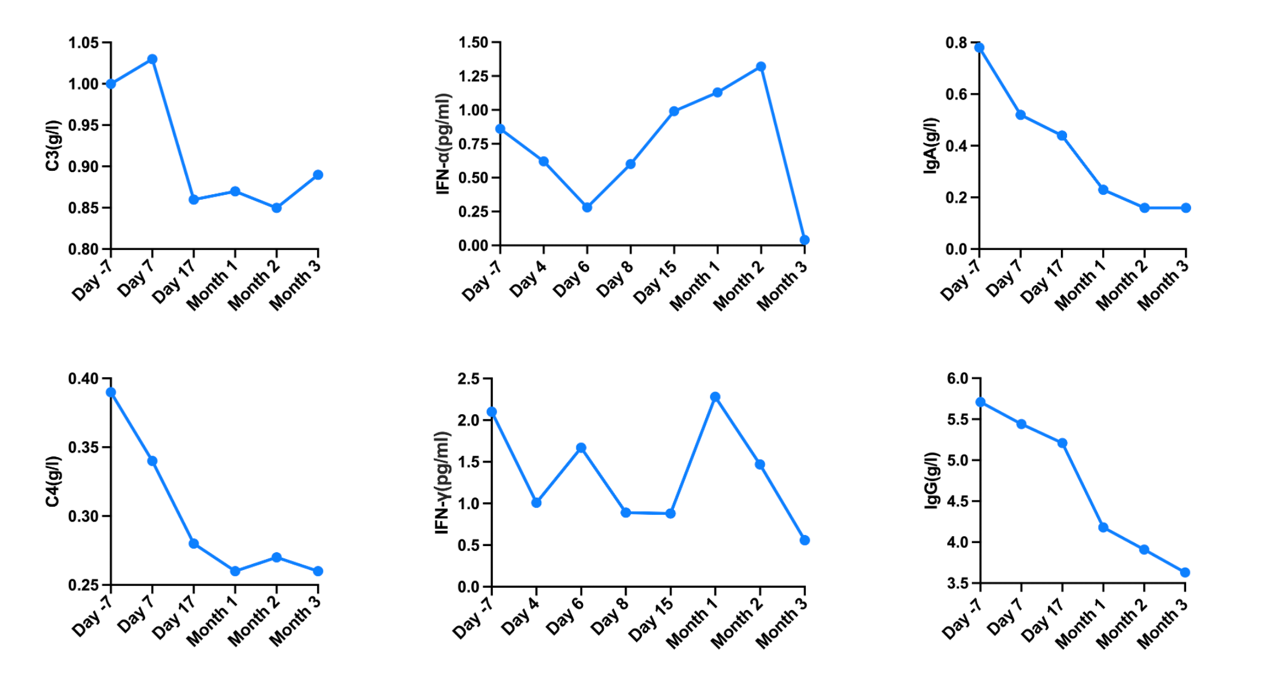
**

Figure S1. Complement component 3, complement component 4, interferon-α, interferon-γ, immunoglobulin A, immunoglobulin G levels before and after CAR T-cell infusion.
