# Supplementary material for: Treatment of pro-B acute lymphoblastic leukemia and severe plaque psoriasis with anti-CD19 CAR T cells: a case report
Source: Front Immunol. 2025 Mar 3;16:1529745. doi: 10.3389/fimmu.2025.1529745 (PMC11911368; doi:10.3389/fimmu.2025.1529745)
Supplement: Supplementary file 2 [file Table1.docx]

1. **Supplementary tables.** Chemotherapy schedule and response before CAR T cell therapy.

| Date | Chemotherapy | | | | Response | |
| --- | --- | --- | --- | --- | --- | --- |
| 2023.11.8-2024.12.5 | Vincristine  Daunorubicin  Cyclophosphamide  Pegaspargase  Prednisone  Venetoclax | 2mg  60/40mg  2.2g  3750U  60/30mg  100/200/400mg | d1,8,15,22  d1-2/3  d1,15  d4  d1-14/15-28  d6/7/8-14 | | CR with MRD  MFC-MRD: 0  NGS-MRD:1.8 x10^-4^ | |
| 2023.12.20-2023.12.28 | Cyclophosphamide  Cytarabine  Mercaptopurine  Vindesine  Pegaspargase  Venetoclax | 1.8g  200mg  100mg  2mg  3750U  400mg | d1  d1-2; d8-9  d1-7  d1  d5  d1-7 | | CR with MRD  MFC-MRD: 0.01%  NGS-MRD:3.79x10^-4^ | |
| 2024.2.2-2024.2.10 | Cyclophosphamide  Cytarabine  Mercaptopurine  Vindesine  Pegaspargase  Venetoclax | 1.8g  200mg  100mg  2mg  3750U  400mg | d1  d1-2; d8-9  d1-7  d1  d5  d1-7 | CR with MRD  MFC-MRD: 0.02%  NGS-MRD:3.16x10^-4^ | |  |
| 2024.3.27-2024.4.6 | Methotrexate  Vindesine  Pegaspargase  Venetoclax | 5.5g  4mg  3750U  400mg | d1  d1  d3  d5-11 | / | |  |
| 2024.4.15-2024.4.18 | Methotrexate  Vindesine  Pegaspargase | 5.5g  4mg  3750U | d1  d1  d3 | CR with MRD  MFC-MRD: 0  NGS-MRD:1.32 x10^-5^ | |  |

CR: Bone marrow blasts <5%; absence of circulating blasts; absence of extramedullary disease; ANC ≥1.0×10^9^/L (1,000/mL); platelet count ≥100×10^9^/L (100 000/mL).
